# Pazopanib in Patients with Clear-Cell Renal Cell Carcinoma: Seeking the Right Patient

**DOI:** 10.3389/fphar.2017.00329

**Published:** 2017-06-21

**Authors:** Camillo Porta, Alessandra Ferrari, Anna M. Czarnecka, Cezary A. Szczylik

**Affiliations:** ^1^Medical Oncology, San Matteo University Hospital Foundation (IRCCS)Pavia, Italy; ^2^Italian Group of Onco-Nephrology (GION)Pavia, Italy; ^3^Department of Oncology with Laboratory of Molecular Oncology, Military Institute of MedicineWarsaw, Poland

**Keywords:** angiogenesis inhibitor, clear cell renal carcinoma, pazopanib, real-world, poor risk

## Introduction

Since its early development, the vascular endothelial growth factors (VEGFRs) inhibitor pazopanib showed both activity and tolerability in patients with metastatic RCC (mRCC) (Hurwitz et al., 2009), results which were subsequently confirmed in the following studies. A phase II study was at first designed as a randomized discontinuation study, but lately revised to an open-label study based on week 12 response rate (RR) of 38% in the first 60 patients. Thus, the primary end-point was changed from progressive disease rate at 16 weeks post-randomization to RR (Hutson et al., 2010). Overall RR was 35%, median duration of response was 68 weeks, and median progression-free survival (PFS) was 52 weeks (Hutson et al., 2010).

Pazopanib was registered based on a global randomized, placebo-controlled, phase III trial. Of the 435 patients enrolled, 233 (54%) were treatment-naïve, while 202 (46%) were pre-treated with cytokines. Pazopanib significantly prolonged PFS in overall study population [median PFS: 9.2 vs. 4.2 months, hazard ratio (HR): 0.46, 95% CI: 0.34–0.62, *p* < 0.0001], in the cytokine-pretreated subpopulation, as well as in the treatment-naïve subpopulation (median PFS: 11.1 vs. 2.8 months, HR: 0.40, 95% CI: 0.27–0.60, *p* < 0.0001), compared to placebo (Sternberg et al., 2010). Furthermore, the objective response rate (ORR) was 30% with pazopanib with a median duration of response longer than 1 year. None clinically important difference in quality of life for pazopanib vs. placebo was observed in the safety analysis (Sternberg et al., 2010).

More recently, the phase III COMPARZ study directly compared pazopanib with sunitinib in a non-inferiority study (Escudier et al., 2014). Pazopanib was non-inferior to sunitinib in terms of PFS (HR: 1.05, 95% CI: 0.90–1.22—predefined upper bound of the 95% CI < 1.25), and similar in OS (HR: 0.91, 95% CI: 0.76–1.08; Motzer et al., 2013); ORR was higher for pazopanib, as compared to sunitinib (31 vs. 25%, *p* = 0.03; Motzer et al., 2013). In terms of safety, pazopanib-treated patients experienced less fatigue, fewer side effects, including soreness of hand/foot and mouth/throat, and were more satisfied with treatment than those who received sunitinib (Motzer et al., 2013; Table 1). Less fatigue and better overall quality of life were the most common reasons that justified the preference of pazopanib vs. sunitinib (70 vs. 22%, *p* < 0.01) in the PISCES study, a cross-over, double blind trial which specifically assessed the innovative endpoint of patients' preference (Escudier et al., 2014).

**Table 1:** Comparison of baseline characteristics, clinical outcomes and adverse events in pivotal studies.

	Baseline characteristics^*^	**Clinical outcomes (95% CI)**	**Adverse events^§^ (% grade 3/4)**
Hutson et al., 2010	Median age 59.8 years Clear cell and predominantly clear cell 100% Median time since diagnosis 223 days ECOG ≤ 1 100% MSKCC Poor 2% Prior nephrectomy 91%	PFS 52 (44–60) weeks ORR 34.7%	Diarrhea (4%), hypertension (9%), hair depigmentation (−), nausea (<1%), anorexia (<1%), vomiting (<1%), fatigue (5%), asthenia (3%), abdominal pain (2%), headache, dysgeusia, cough, abdominal pain (3%), rash (<1%), constipation (<1%), arthralgia (<1%), back pain (<1%), dizziness (<1%), hand-foot syndrome (2%), dyspepsia (1%), alopecia (−), peripheral edema (−)
			Clinical chemistry ALT elevation (9%), AST elevation (6%), bilirubin elevation (<1%), hyponatremia (8%), hyperkalemia (4%), alkaline phosphatase elevation (2%), anemia (2%), creatinine elevation, lipase increased (10%), amylase increased (3%)
			Hematologic Leukopenia (1%), neutropenia (3%), thrombocytopenia (2%), lymphocytopenia (10%)
Sternberg et al., 2010	Median age 59 years Clear cell and predominantly clear cell 100% Median time since diagnosis 15.7 months ECOG ≤ 1 100% MSKCC Poor 9% Prior nephrectomy 89%	PFS 9.2 months ORR 30% (25.1–35.6%)	Diarrhea (3%), hypertension (4%), hair depigmentation (<1%), nausea (<1%), anorexia (2%), vomiting (2%), fatigue (2%), asthenia (3%), abdominal pain (2%), headache (−)
			Clinical chemistry ALT elevation (12%), AST elevation (7%), hyperglycemia (<1%), bilirubin elevation (3%), hypophosphatemia (4%), hyponatremia (5%), hypocalcemia (5%), hypomagnesemia (3%)
			Hematologic Leukopenia, neutropenia (1%), thrombocytopenia (<1%), lymphocytopenia (4%)
Motzer et al., 2013	Median age 61 years Clear cell and predominantly clear cell 100% KPS > 70 100% Prior nephrectomy 82% Lactate dehydrogenase ≤ 1.5 ULN 93% MSKCC Poor 12%	PFS 8.4 (8.3–10.9) months ORR 31% (26.9–34.5%)	Diarrhea (9%), hypertension (15%), hair depigmentation (−), nausea (2%), anorexia (1%), vomiting (2%), fatigue (10%), asthenia (3%), abdominal pain (2%), headache (3%), dysgeusia (<1%), cough, abdominal pain (2%), rash (1%), constipation (1%), arthralgia (<1%), back pain (1%), dizziness (1%), hand-foot syndrome (6%), dyspepsia, alopecia, peripheral edema, proteinuria (4%), weight loss (1%), stomatitis (1%), hypothyroidism (−), mucosal inflammation (−)
			Clinical chemistry ALT elevation (17%), AST elevation (12%), bilirubin elevation (3%), hyperglycemia (5%), hypophosphatemia (4%), hyponatremia (7%), hypocalcemia (<1%), hypoalbuminemia (<1%), hyperkalemia (3%), alkaline phosphatase elevation (3%), anemia (1%), creatinine elevation (<1%), hypoglycemia (<1%), hypokalemia (2%), hypermagnesemia (2%)
			Hematologic Leukopenia (1%), neutropenia (4%), thrombocytopenia (3%), lymphocytopenia (5%)

* Baseline characteristics of patients treated with pazopanib;

§ *Adverse events with incidence >10%, the cumulative incidence of grade 3 and 4 adverse events is reported between parentheses, (−) none events of grade 3 and 4; ECOG, Eastern Cooperative Oncology Group; KPS. Karnofsky Performance Status; MSKCC, Memorial Sloan-Kettering Cancer Center; IMDC, International Metastatic Renal Cell Carcinoma Database Consortium; CI, confidence interval; PFS, progression free survival; OS, overall survival; ORR, overall response rate (complete response + partial response); ALT, alanine aminotransferase; AST, aspartate aminotransferase*.

## Who is the right patient for pazopanib?

Pazopanib, as well as other targeted therapies, has been approved based on results from clinical trials which have strict inclusion criteria to guaranty safety and maintain internal validity and patient homogeneity (Booth and Tannock, 2014). In pivotal and COMPARZ trials, pazopanib was mainly tested in patients with good ECOG performance status (PS) and good and intermediate features according to the MSKCC prognostic classification (Motzer et al., 2002). Data from daily clinical practice on a wider patients' population could complete the picture on pazopanib activity and would help to identify the patient who may have major benefits from pazopanib treatment. Theoretically, the experience with pazopanib from clinical trials could be more easily mirrored into “good fit” patients who are more similar to those enrolled in pivotal studies. However, due to its favorable tolerability profile, pazopanib therapy is frequently considered also for patients with suboptimal features, such as elderly, frail patients, and those at poor risk.

Based on these considerations, who could be considered the “right” patient for pazopanib treatment?

## How to match clinical trial evidence with real-world data

A retrospective analysis of International mRCC data-base consortium (IMDC) data indicated that 35% of patients with mRCC were trial ineligible (Heng et al., 2014). The comparison between eligible and ineligible patients showed that the ineligible group had a poorer prognostic profile and less frequently underwent nephrectomy; clinical outcomes were also worse in ineligible patients, with lower response rate and shorter PFS and OS (Heng et al., 2014). Real-world studies may offer a more realistic picture of clinical outcomes in unselected patients with mRCC, including patients with co-morbidities, brain metastases, histologies different from typical clear cell, or elderly (Mao and Rini, 2014). Taken together, data from formal clinical trials and real-world studies confirmed the efficacy of pazopanib in prolonging PFS and OS in “fit” patients, but also provided evidence about potential benefits achievable in difficult subjects (Ruiz-Morales et al., 2016).

## Young and fit patients

Although, mRCC is frequently observed in 60–70 years old patients, both clinical trials, as well as real-world studies included patients as young as 25 years-old (phase III trial). Noteworthy in the same study, the median age was 59 years. A subgroup analysis of the phase III trial showed the same HR for PFS for patients aged over or below 65 years (Sternberg et al., 2010). On the contrary, in the COMPARZ trial, a pre-defined subgroup analysis indicated a trend toward a longer PFS for patients younger than 65 years old and for patients with a Karnofsky performance status (KPS) between 90 and 100% (Motzer et al., 2013).

In real-world studies, the median age was higher, although young patients were even included. In the MD Anderson Cancer Center cohort, the age ranged from 34 to 91 years, while in the US Oncology Network population, just 5% of patients were younger than 50 years, and 40% younger than 65 years (Vogelzang et al., 2015; Matrana M. et al., 2016). In the latter study, PFS for patients with a baseline ECOG PS = 0 was significantly longer than that of patients with ECOG PS = 1 (11.1 vs. 7.1 months, *p* < 0.007; Vogelzang et al., 2015). Furthermore, in the UK retrospective Christie's study (Galvis et al., 2013), pazopanib-treated patients aged < 60 years had longer median OS compared with patients aged 61–70 or >70 years. Finally, the retrospective analysis conducted by IMDC on more than 7,000 patients with mRCC treated with either pazopanib or sunitinib showed a similar HR for PFS irrespective of KPS (> or < 80%; Ruiz-Morales et al., 2016). Beyond these data, pazopanib favorable safety profile makes it a potentially ideal treatment for patients still in their working years, when severe treatment-toxicities may greatly hamper their lifestyle.

## Patients with good or intermediate prognostic features

In pivotal clinical trials of almost all targeted agents, these agents had been tested in patients with good or intermediate MSKCC risk, who underwent cytoreductive nephrectomy before antiangiogenic therapy (89–91%; Hurwitz et al., 2009; Hutson et al., 2010). In pivotal studies, pazopanib efficacy on PFS was independent from MSKCC stratification (as well as from age and ECOG PS; Hurwitz et al., 2009; Hutson et al., 2010). Similarly, in the COMPARTZ study, pazopanib was non-inferior compared to sunitinib, regardless of MSKCC risk factors (Motzer et al., 2013).

Consistently, in the Christie's study, patients with good risk (per both MSKCC and IMDC) had a PSF of 29 months, whereas OS at the time of the analysis was not reached (median OS, 19 months; Galvis et al., 2013). In the MD Anderson Cancer Center cohort, the MSKCC good risk group had a median PFS of 21.1 and OS of 35.4 months; similar results were obtained with IMDC model (Matrana M. et al., 2016). In the SPAZO study, patients with favorable IMDC risk had PFS of 32.4 months and 81.6% of patients survived during 2-years follow-up with a longer OS (not reached), as compared to those at intermediate (21.6 months) or poor risk (7.1 months) (Pérez-Valderrama et al., 2016).

## Poor risk patients

Approximately, 20–30% of mRCC patients are categorized as poor risk according to either the MSKCC or the IMDC criteria (Porta et al., 2016). In the COMPARZ study, 12 and 19% of patients was defined as at poor-risk, per MSKCC and IMDC criteria, respectively (Motzer et al., 2013), whereas the phase III trial included only 3% of patients with poor MSKCC risk features (Sternberg et al., 2010). In these studies, pazopanib improved PFS or was not-inferior vs. sunitinib, regardless of prognostic classification. Several real-world studies specifically highlighted clinical outcomes of poor risk patients, after stratifying for prognostic risk (Galvis et al., 2013; Kim et al., 2016; Matrana M. et al., 2016; Pérez-Valderrama et al., 2016). As expected, poor risk patients had less benefit from treatment, compared with those with good or intermediate features. For example, in the SPAZO study poor risk patients (23.4% of the whole patient population) had a lower PFS (4 months) and OS (7.1 months) compared to the overall population (Pérez-Valderrama et al., 2016). In a South Korean retrospective review of 172 mRCC patients with poor risk features (per the modified MSKCC criteria used in the Temsirolimus phase III trial), pazopanib was significantly more effective than sunitinib in terms of PFS (9.8 vs. 4.3 months, *p* = 0.04; Kim et al., 2016). This study was a retrospective analysis and include Asian patients who frequently reported hematologic toxicities, hypertension, and hand-foot syndrome (Kim et al., 2016). However, pazopanib safety profile makes it a reasonable option for patients who have a narrow benefit to harm ratio.

## Patients needing tumor shrinkage

Traditionally, sunitinib is consider the most potent VEGFR-inhibitor on the market, but available data clearly show that also pazopanib can induce a high percentage of ORR. In the phase III study, ORR was 30% in the overall population and 32% in the treatment-naïve patients (Sternberg et al., 2010). Furthermore, the COMPARZ trial showed a significantly higher ORR with pazopanib than with sunitinib (31% vs. 25%, *p* = 0.03; Motzer et al., 2013). The activity of pazopanib was also confirmed in the real-world setting: in the SPAZO trial, the ORR in the overall population was 30.3% (Pérez-Valderrama et al., 2016) and the activity was dependant on patients' general conditions (ORR 44% in IMDC good risk patients vs. 17.3% in poor risk; Pérez-Valderrama et al., 2016). These data suggest a preeminent role of pazopanib in patients needing tumor shrinkage, differently from what perceived to date (Rothermundt et al., 2015).

## Patients with sarcomatoid RCC

Sarcomatoid transformation is present in 5% of RCC at diagnosis and may occur in all RCC histological subtypes, no longer representing a distinct histological entity (Bellmunt et al., 2014). Sarcomatoid renal cell carcinoma (sRCC) is defined as any histological RCC type containing foci of high-grade malignant spindle cells (Lopez-Beltran et al., 2006). It has been shown that even a low number of cells with sarcomatoid de-differentiation may be clinically relevant and, therefore, should be included (and possibly quantified) in the pathology report (Lopez-Beltran et al., 2006). Not only sRCC is associated with a significantly poor PFS and OS after adjusting for individual IMDC risk factors (HR = 1.5; *p* < 0.0001) (Kyriakopoulos et al., 2015), but sarcomatoid de-differentiation ≤ 40% (Park et al., 2016) or < 20% (Golshayan et al., 2009) (*p* < 0.001) were also identified as independent prognostic factors affecting PFS of patients treated with tyrosine kinase inhibitors. Best response to VEGFR-TKIs was achieved in patients with up to 10% sarcomatoid component (Park et al., 2014). Pazopanib was active in patients with sRCC, irrespective of the predominant histological subtype (Matrana et al., 2011; Beuselinck et al., 2014; Costello et al., 2014; Matrana M. R., et al., 2016; Stukalin et al., 2017). A recent meta-analysis on therapies used to treat non-clear cell RCC confirmed pazopanib activity in sRCC, not lower than that observed in clear cell RCC (Vera-Badillo et al., 2014).

## Caveats: hepatotoxicity and cardiotoxicity

Drug-induced liver chemistry abnormalities, primarily transaminase elevations, are commonly observed in pazopanib-treated patients. Most of transaminase elevations were isolated and asymptomatic, occurred 3–9 weeks after initiation of therapy (median 42 days) and resolved within a median time of 30 days following onset (Powles et al., 2015). However, potential liver toxicity of pazopanib may influence the decision-making process on patient selection and monitoring. Recently, a model based on baseline patient's characteristics (age, gender, race, hypertension, performance status, tumor type, prior antineoplastic treatment, concomitant treatment with CYP450, P-gp, or breast cancer resistance protein inhibitors, weight, alanine aminotransferase, aspartate aminotransferase, alkaline phosphatase, and bilirubin) seemed to predict the liver toxicity risk with reasonable accuracy (Kattan et al., 2017). After further validation, this tool may provide a helpful aid to personalize pazopanib therapy on single patient.

Cardiotoxicity has been frequently observed with multi-targeted VEGFR tyrosine kinase inhibitors, but it does not seem to be a pharmacologic class-wide effect of VEGFR inhibition. Few data are currently available, but evidence suggests a favorable cardiovascular profile. Some cautions should be taken in patients with low heart rate at baseline (< 60 bpm), a history of syncope or arrhythmia, sick sinus disease, or congestive heart failure (Heath et al., 2013).

## Conclusions

Current guidelines suggest pazopanib as one of the standards of care for first-line treatment of mRCC; in the presence of just one head-to-head comparison, which demonstrated the non-inferiority of pazopanib as compared to sunitinib, it is crucial to figure out the ideal patients to treat with one or the other agent.

Although, its favorable safety profile makes pazopanib appealing for treatment of frail patients, elderly, and those with poor risk prognostic features, available data promote its use in young and fit patients, with good performance status and good prognostic features. Furthermore, its ability to induce significant tumor shrinkage is emerging in recent years, thus supporting its potential in patients who need tumor shrinkage.

## Author contributions

CP, AF, AMC, CAS, contributed to analyze literature and revise for critical intellectual content. CP is accountable for all aspects of the work to ensure accuracy and integrity. Authors approved the paper.

### Conflict of interest statement

CP is a consultant for Novartis, Pfizer, BMS, Ipsen, Eisai, Jannsen, EUSA, Peloton, speaker for Novartis, Pfizer, BMS, Ipsen, Eisai, Jannsen, and received Research funds from Pfizer. The other authors declare that the research was conducted in the absence of any commercial or financial relationships that could be construed as a potential conflict of interest.
